# Race modifies the relationship between cognition and Alzheimer’s disease cerebrospinal fluid biomarkers

**DOI:** 10.1186/s13195-017-0315-1

**Published:** 2017-11-02

**Authors:** Jennifer C. Howell, Kelly D. Watts, Monica W. Parker, Junjie Wu, Alexander Kollhoff, Thomas S. Wingo, Cornelya D. Dorbin, Deqiang Qiu, William T. Hu

**Affiliations:** 1 Department of Neurology, Emory University School of Medicine, 615 Michael Street, 505F, Atlanta, GA 30322 USA; 2 Center for Neurodegenerative Diseases, Emory University School of Medicine, Atlanta, GA USA; 3 Alzheimer’s Disease Research Center, Emory University School of Medicine, Atlanta, GA USA; 4 Department of Radiology, Emory University School of Medicine, Atlanta, GA USA

**Keywords:** African American, Amyloid, Dementia, Endothelial dysfunction, Mild cognitive impairment, Tau

## Abstract

**Background:**

African Americans have been reported to have a higher prevalence of Alzheimer’s disease (AD) than Caucasians, but etiology-specific AD biomarkers have not been systematically analyzed in older African Americans. Coexisting cerebrovascular disease may also contribute to this increased prevalence. We hypothesized that cerebrospinal fluid (CSF) biomarkers of amyloid, neurodegeneration, and endothelial dysfunction would differ between older African Americans and Caucasians with normal cognition and cognitive impairment associated with AD.

**Methods:**

We prospectively recruited 135 older Americans to undergo detailed clinical, neuropsychological, genetic, magnetic resonance imaging (MRI), and CSF analysis from 2013 to 2015 at Emory University (Atlanta, GA, USA). We compared levels of CSF markers for β-amyloid (Aβ42, Aβ40), total and phosphorylated tau (t-tau and p-tau_181_, respectively), endothelial dysfunction (soluble vascular cell adhesion molecule 1, soluble intercellular adhesion molecule 1), α-synuclein, and neurodegeneration (neurofilament light chain [NfL]), as well as MRI markers, for hippocampal atrophy and cerebrovascular disease (white matter hyperintensity [WMH] volume).

**Results:**

Sixty-five older African Americans (average age, 69.1 years) and 70 older Caucasians (average age, 70.8 years) were included. After adjusting for demographic variables, AD risk alleles, and cognitive function, older African Americans had lower CSF levels of p-tau_181_ (difference of 7.4 pg/ml; 95% CI, 3.7–11.2 pg/ml; *p* < 0.001), t-tau (difference of 23.6 pg/ml; 95% CI, 9.5–37.7; *p* = 0.001), and Aβ40 (difference of 1.35 ng/ml; 95% CI, 0.29–2.42 ng/ml; *p* = 0.013) despite similar levels of Aβ42, NfL, WMH volume, and hippocampal volume. Cognitively impaired African Americans also had lower CSF t-tau/Aβ42 (difference of 0.255 per 1-SD change in composite cognition; 95% CI, 0.100–0.409; *p* = 0.001) and p-tau_181_/Aβ42 (difference of 0.076 per 1-SD change in composite cognition; 95% CI, 0.031–0.122; *p* = 0.001). These could not be explained by measured biomarkers of non-AD processes, but African Americans may be more susceptible than Caucasians to the cognitive effects of WMH.

**Conclusions:**

Despite comparable levels of CSF Aβ42 and Aβ42/Aβ40, cognitive impairment in African Americans is associated with smaller changes in CSF tau markers but greater impact from similar WMH burden than Caucasians. Race-associated differences in CSF tau markers and ratios may lead to underdiagnosis of AD in African Americans.

**Trial registration:**

ClinicalTrials.gov, NCT02089555. Retrospectively registered on 14 March 2014.

## Background

Multiple studies have shown quantitative and qualitative differences in Alzheimer’s disease (AD) between African Americans and Caucasians. At the population level, the prevalence of AD dementia is nearly doubled in African Americans compared with Caucasians [1, 2]; African Americans are more likely than Caucasians to have multiple members with dementia within the same family [3]; and genome-wide association studies have identified genetic variants uniquely associated with AD in African Americans (e.g., *ABCA7* [4–6], *COBL* [7]). Cognitively, African Americans with AD are more likely than Caucasians to have nonamnestic profiles [8] and slower decline [9]. At autopsy, African Americans with AD also have greater ischemic and Lewy body copathology and perhaps less transactive response DNA-binding protein 43 kDa (TDP-43) pathology [10, 11]. Together, these findings point to a possible AD endophenotype in African Americans, although few epidemiologic or genome-wide association studies have addressed the accuracy of clinical AD diagnosis. This is especially relevant if a greater proportion of African Americans than Caucasians with vascular dementia are misdiagnosed with AD, which can potentially be corrected through antemortem biomarkers associated with amyloid, tau, and vascular pathology. However, African American recruitment into modern AD biomarker studies such as the Alzheimer’s Disease Neuroimaging Initiative (ADNI) has traditionally been limited [12], and there are few longitudinal clinicopathologic studies involving African Americans that have provided autopsy-based information.

We hypothesized that etiologic biomarkers for AD such as cerebrospinal fluid (CSF) protein profiles can improve the antemortem characterization of AD and non-AD pathology in older African Americans [13, 14]. Because of interindividual heterogeneity in cognition, genetic risks, and comorbidities, enrolling African Americans at their population prevalence (~13%) is likely insufficiently powered to detect race-associated differences in most biomarker studies. Therefore, we prospectively recruited similar numbers of older African Americans and Caucasians with normal cognition, mild cognitive impairment (MCI), and AD dementia to undergo detailed clinical, neuropsychological, magnetic resonance imaging (MRI), genetic, and CSF analysis [15]. We characterized the cohort’s CSF amyloid (β-amyloid 1–40 and 1–42 [Aβ40 and Aβ42, respectively]), tau (total tau and tau phosphorylated at threonine 181 [t-tau and p-tau_181_, respectively]), neurodegenerative (neurofilament light chain [NfL]), and candidate endothelial markers (soluble vascular cell adhesion molecule 1 [sVCAM-1]; soluble intercellular cell adhesion molecule 1 [sICAM-1]) [16, 17] to test their associations with cognitive impairment within each race.

## Methods

### Ethics, consent, and permission

This study was approved by the Emory Institutional Review Board. Informed consent was obtained from all subjects or their authorized representatives.

### Subjects

We enrolled 65 older African Americans and 70 older Caucasians into a cross-sectional study at Emory University from 1 July 2013 to 30 June 2015. Based on our preliminary data, this sample size was sufficient to detect a difference in CSF t-tau levels. Recruitment for this study was previously described [15]. Briefly, potential participants were identified through the Emory Alzheimer’s Disease Research Center, the Emory Cognitive Neurology Clinic, or community forums on aging and memory, and individuals were contacted if there was a subjective report of normal cognition or a diagnosis of MCI or AD dementia. Study protocols, including CSF and MRI analysis, were discussed with potential participants, and those diagnosed with non-AD dementia (e.g., vascular, dementia with Lewy bodies, frontotemporal dementia or primary progressive aphasia, normal pressure hydrocephalus) were excluded from the study. Subjects with MCI with features suggestive of non-AD pathology (e.g., rapid eye movement sleep behavior disorder) were also excluded. A total of 288 subjects were contacted, and 135 (47%) consented to participate. The study completion rate was 98% for MRI (133 of 135) and 93% for lumbar puncture (126 of 135). Subjects who did not undergo lumbar puncture were similar to those who did with regard to age, sex, race, education, and apolipoprotein E (*APOE*) and *ABCA7* genotypes. All biochemical and MRI analyses were performed by experienced operators blinded to the subject’s race and cognitive functioning.

### Clinical and neuropsychological characterization

Each participant underwent a detailed interview so that we could obtain demographic information, including self-reported race (Caucasians of Hispanic or Latino ethnicity were not included in this study), vascular risk factors (coronary artery disease, congestive heart failure, atrial fibrillation, hypertension, hyperlipidemia, diabetes, suspected transient ischemic attack), other medical comorbidities (e.g., cancer), and medications (e.g., use of angiotensin-converting enzyme inhibitors or angiotensin II receptor blockers). Each subject also underwent a detailed neurologic examination and neuropsychological analysis for assessment of function in cognitive domains. These included (1) memory (Consortium to Establish A Registry for Alzheimer’s Disease word list delayed recall, Brief Visual Memory Test–Revised [BVMT-R] delayed recall), (2) executive function (Trail Making Test B, reverse digit span [RD], Symbol Digit Substitution Test, and letter-guided fluency), (3) language (Boston Naming Test [60 items], category fluency), and (4) visuospatial function (Judgment of Line Orientation [JOLO], Rey-Osterrieth complex figure test). With the exception of BVMT-R, JOLO, and RD, subtest Z-scores were calculated according to published normative data, adjusting for age, sex, education, and race [18, 19]. Domain-specific Z-scores were calculated by averaging subtest Z-scores, and Z-scores for the four domains were averaged to generate composite cognitive Z-scores. This approach was used in previous longitudinal multiracial studies, including ADNI, and it allows for domain-specific and global assessment of cognition [20–23]. Each participant was then assigned a diagnosis according to consensus criteria (Table 1), including those for normal cognition, MCI [24, 25], and AD dementia (global Clinical Dementia Rating 1 or 2) [26, 27]. Linear regression analysis among subjects with normal cognition showed that BVMT-R scores were not affected by race (*p* = 0.207). African American subjects’ performance on JOLO was significantly better than published norms [28], so a regression analysis was used to develop Z-scores for African Americans. This showed that JOLO scores correlated with sex, race, and education (4.05 × sex [1 = male; 0 = female] + 0.60 × years of education + 1.94 × race [1 = Caucasian; 0 = African American]). RD scores were also influenced by race (1.297 × race [1 = Caucasian; 0 = African American]). Mean and SD values among subjects with normal cognition were then calculated after adjusting for these variables to obtain Z-scores. Cognitively impaired subjects suspected of having a non-AD dementia (vascular, Lewy body, and frontotemporal dementia) were excluded. Because we aimed to examine the relationship between race and cognitive function and the sensitivity of CSF t-tau/Aβ42 > 0.39 for AD derived from a previous autopsy study [29] is not 100%, we did not exclude MCI and AD subjects whose CSF t-tau/Aβ42 ratio was < 0.39.

**Table 1:** Baseline characteristics of African American and Caucasian research participants

	Overall		Normal cognition		MCI		AD	
	African American (*n* = 65)	Caucasian (*n* = 70)	African American (*n* = 27)	Caucasian (*n* = 29)	African American (*n* = 27)	Caucasian (*n* = 25)	African American (*n* = 11)	Caucasian (*n* = 16)
Age, years (SD)	69.1 (7.4)	70.8 (7.7)	67.5 (6.2)	71.4 (8.1)	67.7 (7.6)	71.5 (5.8)	71.4 (9.1)	68.5 (9.4)
Male sex, *n* (%)	29 (45%)	29 (41%)	10 (37%)	12 (41%)	15 (56%)	10 (40%)	4 (36%)	7 (44%)
Education, years (SD)	16.1 (2.8)	16.4 (2.7)	15.8 (2.7)	17.0 (2.6)	16.3 (2.9)	17.1 (2.6)	16.7 (3.4)	14.2 (1.9)
MMSE (SD)	26.1 (4.0)	26.7 (3.6)	28.0 (1.8)	28.9 (0.8)	26.6 (2.4)	27.6 (1.7)	20.5 (5.9)	21.4 (3.4)
Hypertension	47 (72%)^a^	32 (46%)	17 (63%)	13 (45%)	21 (78%)	14 (56%)	9 (82%)	5 (31%)
Diabetes	22 (34%)^a^	4 (6%)	9 (33%)	2 (7%)	8 (30%)	0	5 (45%)	2 (12%)
Average number of vascular risk factors (SD)	1.9 (1.2)^a^	1.3 (1.1)	1.5 (1.1)	1.3 (1.3)	2.2 (1.3)	1.4 (0.9)	2.2 (1.2)	1.0 (1.0)
Having at least one *APOE* ε4 allele, *n* (%)	31/60 (52%)	35/70 (50%)	7/25 (29%)	11 (38%)	15/26 (58%)	13 (52%)	9/10 (90%)	11 (69%)
Having *ABCA7* risk allele, *n* (%)	28/60 (47%)^a^	16/70 (23%)	12/25 (48%)	8 (27%)	13/25 (52%)	6 (24%)	3/10 (30%)	2 (12%)
Completed LP, *n* (%)	58 (89%)	68 (97%)	23 (85%)	28 (97%)	25 (93%)	25 (100%)	10 (91%)	15 (94%)
CSF								
Aβ42, pg/ml (SD)	212.3 (118)	207.2 (148)	273 (110)	250 (131)	178 (110)	226 (172)	158 (105)	96 (61)
Aβ40, ng/ml (SD)	7.89 (2.92)	9.29 (3.32)	7.21 (1.96)^a^	10.03 (3.65)	7.83 (3.21)	9.38 (3.18)	9.49 (3.61)	7.91 (2.54)
t-Tau, pg/ml (SD)	47.0 (31.1)^a^	71.5 (47.8)	36.3 (12.0)^a^	58.6 (29.0)	46.4 (38.6)	66.6 (38.4)	72.8 (27.5)	103.8 (72.9)
p-Tau_181_, pg/ml (SD)	17.9 (9.3)^a^	25.6 (12.6)	13.8 (5.1)^a^	22.5 (9.8)	18.8 (10.2)	24.0 (12.0)	25.3 (10.3)	34.2 (14.9)
CSF t-Tau/Aβ42 > 0.39	17/58 (29%)	31/68 (46%)	2/23 (9%)	7/28 (25%)	9/25 (36%)	13/25 (52%)	6/10 (60%)	11/15 (73%)
Log_10_(NfL), ng/ml (SD)	2.87 (0.27)	2.97 (0.19)	2.76 (0.23)	2.96 (0.22)	2.87 (0.26)	2.96 (0.15)	3.14 (0.23)	3.02 (0.22)
Log_10_(WMH), cm^3^ (SD)	0.367 (0.453)	0.437 (0.364)	0.238 (0.337)	0.355 (0.365)	0.401 (0.515)	0.483 (0.308)	0.607 (0.466)	0.514 (0.431)

*Abbreviations: Aβ40* β-Amyloid 1–40, *Aβ42* β-Amyloid 1–42, *AD* Alzheimer’s disease, *APOE* Apolipoprotein E, *CSF* Cerebrospinal fluid, *MCI* Mild cognitive impairment, *MMSE* Mini Mental State Examination, *NfL* Neurofilament light chain, *p-Tau*_*181*_ Tau phosphorylated at threonine 181, *t-Tau* Total tau, *WMH* White matter hyperintensity

Values shown are unadjusted

^a^Values or proportions differed between the two races (*p* < 0.005)

### MRI acquisition and analysis

MRI data were acquired using a MAGNETOM Trio, A Tim System 3.0-T scanner (Siemens Healthcare, Erlangen, Germany), including 3D T1-weighted magnetization-prepared rapid acquisition with gradient echo anatomical imaging (repetition time/inversion time/echo time, 2300/900/3.0 milliseconds; flip angle, 9 degrees; voxel size, 1 × 1 × 1.2 mm^3^; 176 slices). Cortical and subcortical volumes were extracted by employing a user-independent parcellation process in FreeSurfer (version 5.03; Massachusetts General Hospital/Harvard University, Boston, MA, USA) running on the CentOS 6.6 operating system [30]. Subcortical white matter and deep gray matter structures are parcellated by combining data from voxel intensity, probabilistic atlas locations, and local relationships between anatomical structures. Total white matter hyperintensity (WMH) volume was derived from T2-weighted fluid-attenuated inversion recovery sequences as previously described [31]. WMH volume was not normally distributed and was therefore log-transformed for statistical analysis.

### CSF collection and analysis

CSF samples were collected between 8:00 a.m. and 2:00 p.m. without requiring fasting. This window was chosen because CSF Aβ42 levels during these times represent approximately 95–105% of average Aβ42 over time [32]. CSF was immediately aliquotted after collection and before freezing, and otherwise we used the ADNI biofluid protocols, including the use of 24-gauge Sprotte needles, aspiration syringes, and transfer into 15-ml polypropylene tubes. To avoid measurement bias related to well positions, sample positions were randomized for each biomarker assay.

CSF biomarkers were measured following the manufacturers’ protocols: Aβ42, t-tau, and p-tau_18_ levels were measured using the INNO-BIA AlzBio3 immunoassay (Fujirebio, Malvern, PA, USA) in a Luminex 200 platform (Luminex, Austin, TX, USA) [33] (average interplate coefficients of variation of 13% for Aβ42, 10% for t-tau, and 11% for p-tau_181_), Aβ40 levels using the INNOTEST® enzyme-linked immunosorbent assay (ELISA) (Fujirebio), α-synuclein levels using the Novex® ELISA (Thermo Fisher Scientific, Waltham, MA, USA), NfL levels using the NF-light® ELISA (Uman Diagnostics, Umeå, Sweden), and sICAM-1 and sVCAM-1 levels using a commercial multiplex kit (MILLIPLEX® MAP Human Neurodegenerative Magnetic Bead Panel 3 [HNDG3MAG-36 K]; EMD Millipore, Billerica, MA, USA).

### DNA extraction and analysis

DNA was extracted from buffy coat as previously described [34]. Single-nucleotide polymorphism (SNP) genotyping for *APOE* and *ABCA7* was performed using TaqMan® assays (Thermo Fisher Scientific). *ABCA7* SNP rs3764650 (catalogue number 4351379; Thermo Fisher Scientific) was analyzed because of its association with AD [6] and because it is in linkage disequilibrium with SNP rs115550680 implicated in AD in African Americans [4]. Statistical analysis was restricted to those with successful genotyping (*n* = 124 cases; all had CSF, 123 had MRI).

### Statistical analysis

Statistical analysis was performed using IBM SPSS version 22.0 software (IBM, Armonk, NY, USA). Baseline demographic variables and AD risk alleles were compared between African Americans and Caucasians within each diagnostic category (normal cognition, MCI, and AD) using the chi-square test or Fisher’s exact test for categorical variables and Student’s *t* test for continuous variables, with *p* < 0.005 to adjust for multiple comparisons. Among subjects with normal cognition, all biomarkers were normally distributed except for NfL and WMH, which were then log_10_-transformed. Analysis of covariance (ANCOVA) was used to determine race-associated differences in CSF AD biomarkers (t-tau, p-tau_181_, Aβ40, t-tau/Aβ42, p-tau_181_/Aβ42, Aβ42/Aβ40), α-synuclein, and p-tau_181_-to-t-tau ratio (p/t-tau), adjusting first for cognitive functioning (diagnostic category, Mini Mental State Examination [MMSE], or cognitive Z-score). Race and the interaction between race and cognitive function were both included in these models. When appropriate, age, sex, APOE, ABCA7 risk allele status, and CSF Aβ42 levels were further included in the ANCOVA. *F*-statistic and *p* values for main effects were reported, along with coefficient estimate and 95% CI after stepwise removal of factors and covariates with *p* > 0.10. To account for coexisting cerebrovascular risks, hypertension, diabetes, and WMH volume were then added. Furthermore, sVCAM-1 was analyzed first through Pearson’s correlational analysis and then in its own ANCOVA to determine its suitability as a CSF endothelial marker. Finally, because African Americans had lower CSF tau markers than Caucasians but similar WMH volumes, linear regression analysis was used to model potential interactions between race and tau and between race and WMH volume to determine if cognition was more affected in African Americans than in Caucasians on the basis of the same unit of change in CSF tau markers or WMH volume. In this model, composite cognitive Z-score was the dependent variable, with race, sex, having the APOE ε4 allele, having an ABCA7 risk allele, hypertension, and diabetes as categorical variables and age, education, Aβ42, t-tau, log_10_ (WMH), and education as continuous variables. Independent variables were entered in a stepwise fashion to achieve a model consisting of only main effects. Race and education were not significant factors in this model, because the cognitive Z-scores had been adjusted for these two factors, but sex and age remained significant to account for variance from the sex- and age-adjusted cognitive Z-scores. To test the hypothesis that race modified the relationship between cognition and t-tau or WMH, an interaction term of t-tau × race or log(WMH) × race was added to the model.

## Results

### Baseline and genetic characteristics

Among the 135 participants, African Americans were more likely than Caucasians to have the *ABCA7* risk allele (46.7% vs. 22.9%, *p* = 0.005) and the *ICAM1* Lys56Met polymorphism (33.3% vs. 5.7%, *p* < 0.001) (Table 1). African Americans were also more likely to have hypertension (72% vs. 46%, *p* = 0.003) and diabetes (34% vs. 6%, *p* < 0.001) (Table 1). The two groups were otherwise similar in age, sex, education, proportion with the APOE ε4 allele, and other vascular risk factors (Table 1).

### Relationship between race and CSF biomarkers for amyloid and tau

**Fig. 1:**
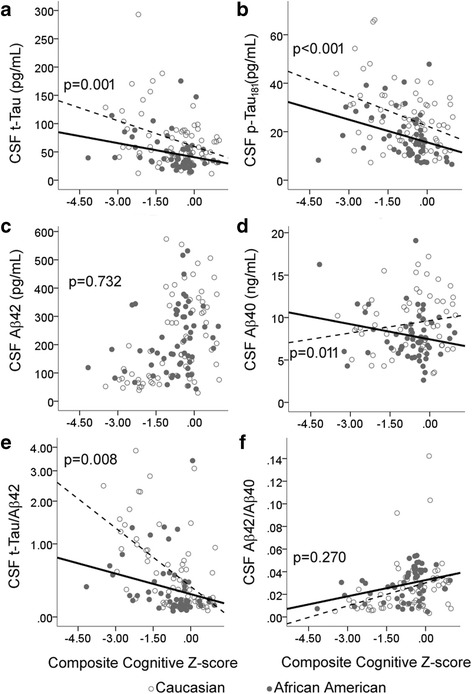
Cerebrospinal fluid (CSF) levels of tau and amyloid markers in older African Americans and Caucasians according to cognitive function. Composite cognitive Z-scores are shown on the *x*-axis (lower score corresponds to worse cognitive function). African Americans (*closed circles*) had lower CSF levels of total tau (t-tau) (**a**), tau phosphorylated at threonine 181 (p-tau_181_) (**b**), and β-amyloid 1–40 (Aβ40) (**d**) than Caucasians (*open circles*). Raw values are shown, with *dashed lines* representing trends among Caucasians and *solid lines* representing trends among African Americans. The differences persisted after adjusting for age, sex, apolipoprotein E (*APOE*) and *ABCA7* genotypes, and β-amyloid 1–42 (Aβ42) levels (**c**), which did not differ between the two groups. CSF biomarker t-tau/Aβ42 ratio was lower in African Americans than in Caucasians when there was cognitive impairment (**e**), but race did not have a significant effect on CSF biomarker Aβ42/Aβ40 (**f**)

As a group (regardless of diagnosis), African Americans had lower CSF levels of p-tau_181_ (17.9 vs. 25.6 pg/ml, *p* < 0.001) (Fig. 1a), t-tau (47.0 vs. 71.5 pg/ml, *p* = 0.001) (Fig. 1b), and Aβ40 levels (7.88 vs. 9.29 ng/ml, *p* = 0.017) (Fig. 1d) than Caucasians, but they had similar Aβ42 levels (Fig. 1c). Univariate analysis of these biomarkers revealed the greatest differences in subjects with normal cognition (Table 1), but the relationship between biomarker levels and demographic variables necessitated the use of ANCOVA to determine whether race influenced CSF biomarker levels in a cognition-dependent or cognition-independent fashion. We found that, independent of cognitive functioning (data for cognitive Z-scores presented; diagnostic category and MMSE produced similar results), age, sex, APOE ε4 and ABCA7 risk alleles, and Aβ42 levels, African Americans had lower levels of t-tau [difference of 23.6 pg/ml; 95% CI, 9.5–37.7; *F*(2,122) = 10.99; *p* = 0.001], p-tau_181_ levels [difference of 7.4 pg/ml; 95% CI, 3.7–11.2 pg/ml; *F*(2,122) = 15.79; *p* < 0.001], and Aβ40 [difference of 1.355 ng/ml; 95% CI, 0.293–2.417 ng/ml; *F*(2,122) = 6.385; *p* = 0.013]. Race also affected the ratio biomarker of t-tau/Aβ42 (Fig. 1e) and p-tau_181_/Aβ42 (not shown) according to cognitive functioning, but it had minimal effect on Aβ42/Aβ40 (Fig. 1f). For both tau markers, cognitively impaired African Americans had lower CSF t-tau/Aβ42 [difference of 0.255 per 1-SD change in composite cognition; 95% CI, 0.100–0.409; *F*(2,122) = 10.67; *p* = 0.001] and p-tau_181_/Aβ42 [difference of 0.076 per 1-SD change in composite cognition; 95% CI, 0.031–0.122; *F*(2,122) = 10.94; *p* = 0.001] ratios than cognitively impaired Caucasians.

### CSF neurodegenerative and endothelial markers do not account for race-associated AD biomarker differences

Lower t-tau and p-tau_181_ levels in African Americans may be explained by misdiagnosis, less neurodegeneration, or greater contribution from non-AD copathology toward cognitive impairment. Because CSF Aβ42 levels were indistinguishable between African Americans and Caucasians, cognitively impaired subjects in the two racial groups were equally likely to have β-amyloidopathy, which makes misdiagnosis less probable. To determine if there was a difference in neurodegeneration between the two races, we examined the relationship between race, CSF NfL levels, and hippocampal atrophy because the latter two are influenced by AD as well as non-AD disorders. ANCOVA showed that cognitively normal African Americans had lower log(NfL) than cognitively normal Caucasians [*F*(2,122) = 3.525; *p* = 0.017], but log(NfL) did not differ between the two races for those with cognitive impairment. Race also had no effect on hippocampal volumes [*F*(2, 122) = 1.78; *p* = 0.185] to suggest less neurodegeneration in cognitively impaired African Americans. Similarly, among those with CSF t-tau/Aβ42 not consistent with AD, race had no effect on CSF α-synuclein levels [implicated in Lewy body disease; *F*(2,75) = 1.078; *p* = 0.303] or p/t-tau ratio [implicated in frontotemporal lobar degeneration with TDP-43 immunoreactive inclusions; *F*(2,75) = 0.775; *p* = 0.382].

**Fig. 2:**
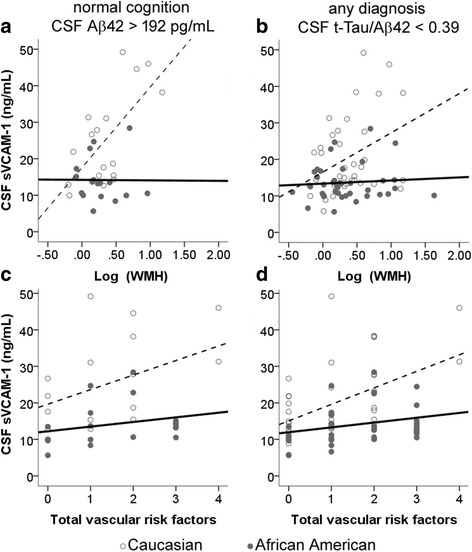
Relationship between cerebrospinal fluid (CSF) soluble vascular cell adhesion molecule 1 (sVCAM-1) levels and other vascular markers according to race. In Caucasians (*open circles*), CSF sVCAM-1 levels strongly correlated with log-transformed white matter hyperintensity (WMH) volumes derived by magnetic resonance imaging (**a**, **b**) and the total number of peripheral vascular risk factors (**c**, **d**) whether a more (**a**, **c**) or less stringent (**b**, **d**) threshold was applied to identify subjects with no Alzheimer’s disease pathology. However, there was no such correlation in African Americans (*closed circles*). *Aβ42* β-Amyloid 1–42, *t-Tau* Total tau

Because African Americans had a higher prevalence of hypertension and diabetes than Caucasians in our cohort (Table 1), cerebrovascular disease could account for cognitive impairment in the setting of lower CSF tau levels. Including hypertension, diabetes, and total WMH volume in the ANCOVA did not change the effect of race on p-tau_181_ [*F*(2,121) = 18.13; *p* < 0.001), t-tau [*F*(2,121) = 6.790; *p* = 0.010], or Aβ40 levels [*F*(2,121) = 5.084; *p* = 0.008]. Therefore, vascular risk factors and total WMH volume do not sufficiently account for the CSF AD biomarker differences between the two races. At the same time, peripheral vascular risk factors may not adequately reflect cerebrovascular disease burden. We thus tested whether CSF levels of two candidate endothelial markers (sVCAM-1 and sICAM-1) could be used as surrogate markers of endothelial dysfunction. As a proof of principle, we first analyzed the relationship between these two markers and race, peripheral vascular risks, and WMH among cognitively normal subjects enriched for those without AD pathology (CSF Aβ42, > 192 ng/ml; *n* = 34). At the univariate level, CSF sVCAM-1 levels, but not sICAM-1 levels, correlated positively with older age (*R* = 0.514; *p* < 0.001), Caucasian race (*R* = 0.35; *p* = 0.026), higher WMH volume (log-transformed; *R* = 0.433; *p* = 0.005), greater number of vascular risk factors (*R* = 0.357; *p* = 0.020), history of congestive heart failure (*R* = 0.444; *p* = 0.003), and history of atrial fibrillation (*R* = 0.399; *p* = 0.009). ANCOVA showed that race modified the relationship between sVCAM-1 levels and WMH volume (Fig. 2a) and between sVCAM-1 levels and vascular risk (Fig. 2b). Similar results were obtained when all subjects with CSF t-tau/Ab42 < 0.39 were included (Fig. 2c, d). In sum, whereas sVCAM-1 correlated with known vascular disease markers in Caucasians, it is a poor measure of endothelial dysfunction in African Americans.

### Race modifies the impact of WMH on cognition

To reconcile our finding that, after controlling for race-adjusted cognitive performance, African Americans had lower CSF tau marker levels than Caucasians despite similar levels of WMH, NfL, and hippocampal volumes, we tested if race modified the relationship between cognition and the two intermediate etiologic biomarkers (tau, WMH). To test this hypothesis, we determined, using multivariate linear regression analysis, whether there was an interaction between race and each of these markers of AD (t-tau, p-tau_181_) or vascular (WMH) pathology to influence cognition (*see* the Methods section above). At the univariate level, cognition was influenced by *APOE* and *ABCA7* genotypes as well as by log_10_(WMH) and CSF levels of Aβ42 and t-tau. Introducing the interaction term of race × log_10_ (WMH) showed that the interaction term had a stronger effect on cognitive Z-scores than log_10_ (WMH) alone (Table 2), and that African American race was associated with greater cognitive impairment than Caucasian race for every unit of change in log_10_ (WMH). This was not the case for tau or p-tau_181_.

**Table 2:** Demographic and biomarker variables that influence cognitive Z-scores

Variables	B (95% CI)	*p* Value
Intercept	−3.08 (−4.70, −1.45)	< 0.001
Log(WMH)	−0.35 (−0.89, 0.19)	0.206
Male sex	0.54 (0.21, 0.87)	0.002
Having at least one *APOE* ε4 allele	−0.36 (−0.71, −0.01)	0.042
Having *ABCA7* risk allele	0.38 (0.04, 0.72)	0.03
Aβ42	0.003 (0.001, 0.004)	< 0.001
t-Tau	−0.006 (−0.010, −0.002)	0.003
Age	0.039 (0.015, 0.063)	0.002
African American race × log(WMH)	−0.496 (−1.074, 0.081)	0.091

*Abbreviations: Aβ42* β-Amyloid 1–42, *APOE* Apolipoprotein E, *t-Tau* Total tau, *WMH* White matter hyperintensity

A stepwise regression model was used to determine factors most strongly associated with cognitive function as reflected by cognitive Z-scores. African American race had a trend of worsening cognitive functioning for the same degree of change in WMH. If the log(WMH) term was removed from this model, African American race was associated with lower cognitive Z-score (by 0.496; *p* = 0.006) per unit change of log(WMH). Race and sex were not significant factors in this model. Similar results were obtained when p-tau_181_ instead of t-tau was entered into the model

## Discussion

Although multicenter efforts such as ADNI have advanced the characterization of AD in terms of cognitive decline, fluid and imaging biomarkers, and genetic variants, whether findings from these homogeneous cohorts can be applied to a more diverse population remains unknown. African Americans represent the largest racial minority group in the United States, and clinicopathologic studies linking their phenotypes to underlying pathology on autopsy or pathology-associated biomarkers are rare. To the best of our knowledge, this is the first study to directly identify race to modify CSF AD biomarker levels and the relationships between WMH and cognition in a small but well-characterized cohort of older African Americans and Caucasians. Whereas race did not affect the primary CSF marker of amyloid deposition (Aβ42 levels), race was associated with lower CSF p-tau_181_ and t-tau levels in African Americans, independent of cognition, and it affected the AD biomarkers t-tau/Aβ42 and p-tau_181_/Aβ42 according to cognitive impairment. Race also affected Aβ40 levels, but it did not influence AD biomarker Aβ42/Aβ40. We propose that these differences did not result from less neurodegeneration or greater endothelial dysfunction in African Americans. Instead, our model suggests that the same degree of WMH had a greater impact on cognition in African Americans than in Caucasians through an as yet unknown mechanism.

Assessing cognitive decline in minority populations has been challenging because of discrepancies between various normative datasets [28, 35, 36]. In keeping with others’ experiences, established normative data underestimated the cognitive functioning of older African Americans in our cohort, possibly owing to their generally high level of education. Objective etiologic biomarkers have the potential of identifying AD and other neurodegenerative diseases early in the disease course, independent of language, cultural, and socioeconomic confounds. Our observation of a race effect on three common AD biomarkers—t-tau, p-tau_181_, and Aβ40—underscores the biological and clinical importance of biomarker studies in minority populations. We believe that cognitive impairment in African Americans was indeed associated with β-amyloidopathy, because CSF Aβ42 levels in subjects with MCI or dementia did not differ between the two races. These findings imply that using existing cutoff values for CSF biomarker levels or ratios involving tau derived from largely Caucasian populations may lack sensitivity in identifying AD in minority populations, and it is not known if cerebral tau imaging can overcome this. We extended these observations by exploring multiple CSF and MRI markers involved in neurodegeneration, and we found race-independent (e.g., NfL) as well as race-dependent (sVCAM-1) markers. However, a key observation was the absence of a difference in CSF Aβ42 levels and WMH volumes between the two races. According to the model of sequential biomarker changes in AD [37], more modest increases in t-tau or p-tau_181_ levels in cognitively impaired African Americans might result in lower CSF NfL levels and greater hippocampal volumes than similarly impaired Caucasians. This was not the case, which led us to search for an alternative explanation for the relatively greater neurodegenerative changes (measured by NfL and hippocampal volumes with less specificity to AD) in African Americans associated with more modest tau changes. Exactly how WMH may result in greater neurodegeneration and cognitive deficits in African Americans is unknown. WMH is thought to represent terminal organ damage from chronic ischemia, and it is possible that African Americans have a greater degree of subclinical endothelial dysfunction than Caucasians. It is also possible that differences in the brain structural (as measured by white matter tract integrity) and functional (as measured by resting state connectivity) networks predispose African Americans to the effects of ischemia. Finally, a synergistic effect between WMH and brain amyloid burden has been reported for cognition [38], and brain amyloid deposition in African Americans may preferentially enhance WMH-associated neurotoxicity over tau-associated neurodegeneration. Future studies should prospectively test each of these hypotheses to identify potentially modifiable factors in an effort to reduce the race-associated increase in AD prevalence [39].

Our model was suggestive of a process involving WMH to account for lower tau marker levels in African Americans, but other explanations need to be considered. There still exists the possibility of greater non-AD pathology in African Americans, because CSF α-synuclein and p/t-tau are imperfect markers of Lewy body and TDP-43 pathology [40]. Although amyloid and tau PET imaging may provide additional information, more detailed neuropathologic characterization may be necessary to definitively determine the AD pathology in African Americans with clinical AD dementia. Variants in the *MAPT* or other genes (besides *APOE* or *ABCA7*) may also influence t-tau levels or the relative levels of different tau isoforms. The H2 haploptype of *MAPT* is rare among African, Asian, and Native American populations [41], but a previous study failed to identify any *MAPT* variant to associate with CSF t-tau or p-tau_181_ levels among those without evidence of brain amyloid deposition [42]. Other limitations of the study include the lack of an independent validation cohort, not using genetic markers to ascertain African ancestry [43] or to account for additional race-related AD risks [7], limited information on environmental exposure, and inclusion of only the most commonly studied AD and endothelial markers. It is also worth emphasizing that genomic African ancestry is inadequate to biologically or sociologically characterize aging and disease in African Americans, as pointed out by many of our participants and colleagues, and this study’s findings may not translate to other African American cohorts of different genetic and socioeconomic backgrounds.

## Conclusions

In sum, we report that CSF tau biomarker levels are strongly influenced by race in a cohort of older African Americans and Caucasians in Atlanta. This has important implications for the use of diagnostic biomarkers derived from CSF and potentially other modalities (e.g., positron emission tomography) in unique populations underrepresented in existing large biomarker studies. Our data also raise the possibility that African American race may modify the relationship between an MRI marker of cerebrovascular disease and cognition, likely through an APOE-independent mechanism. There is an urgent need to test the generalizability of these findings in more cohorts of African Americans.

## Abbreviations

AD: Alzheimer’s disease
ADNI: Alzheimer’s Disease Neuroimaging Initiative
ANCOVA: Analysis of covariance
APOE: Apolipoprotein E
Aβ40: β-Amyloid 1–40
Aβ42: β-Amyloid 1–42
BVMT-R: Brief Visual Memory Test–Revised
CSF: Cerebrospinal fluid
ELISA: Enzyme-linked immunosorbent assay
JOLO: Judgment of Line Orientation
MCI: Mild cognitive impairment
MMSE: Mini Mental State Examination
MRI: Magnetic resonance imaging
NfL: Neurofilament light chain
p-Tau_181_: Tau phosphorylated at threonine 181
RD: Reverse digit span
sICAM-1: Soluble intercellular adhesion molecule 1
SNP: Single-nucleotide polymorphism
sVCAM-1: Soluble vascular cell adhesion molecule 1
TDP-43: Transactive response DNA-binding protein 43 kDa
t-Tau: Total tau
WMH: White matter hyperintensity
